# Comparative Chromosomal Mapping of the 18S rDNA Loci in True Bugs: The First Data for 13 Genera of the Infraorders Cimicomorpha and Pentatomomorpha (Hemiptera, Heteroptera)

**DOI:** 10.3390/genes16121516

**Published:** 2025-12-18

**Authors:** Natalia V. Golub, Boris A. Anokhin, Snejana Grozeva, Valentina G. Kuznetsova

**Affiliations:** 1 Zoological Institute, Russian Academy of Sciences, 199034 St. Petersburg, Russia; natalia.golub@zin.ru (N.V.G.); boris.anokhin@zin.ru (B.A.A.); 2 Institute of Biodiversity and Ecosystem Research, Bulgarian Academy of Sciences, 1000 Sofia, Bulgaria; sngrov@gmail.com

**Keywords:** Heteroptera, Terheteroptera (Cimicomorpha + Pentatomomorpha), karyotypes, 18S rDNA, FISH

## Abstract

**Background/Objectives:** Sites of ribosomal RNA genes are the most widely documented regions of chromosomes in various groups of eukaryotes, including insects. Data on the number and chromosomal location of 45S rDNAs (25S, 5.8S, and 18S rDNA) are actively used to study the diversity of karyotypes, the organization of individual chromosomes, and the evolution of entire genomes. In true bugs (suborder Heteroptera), the number and chromosomal distribution of 18S rDNA loci are currently known for less than 0.5% of described species. Although some patterns of rDNA distribution can already be identified both in individual taxa of true bugs and in the suborder as a whole, there are still negligible data. In order to expand our understanding of the diversity of rDNA distribution in Heteroptera, we studied for the first time the location of 18S rDNA in 13 species from 13 genera (seven families) of the infraorders Cimicomorpha and Pentatomomorpha (=Terheteroptera, the terminal group of Heteroptera). **Methods**: Fluorescence in situ hybridization (FISH) with an 18S rDNA probe was used in our study. **Results**: In total, we have identified three main types of rDNA arrangement: (1) on autosomes, (2) on the X chromosome, and (3) on autosomes and on the X chromosome simultaneously. In most of the studied species, 18S rDNA loci were detected in the terminal position on one pair of autosomes. **Conclusions**: This study contributed to the understanding of the chromosomal distribution of rDNA loci in the infraorders Cimicomorpha and Pentatomomorpha and confirmed the importance of rDNA in the reorganization of the genomes of Heteroptera as a whole.

## 1. Introduction

In higher eukaryotes, ribosomal DNAs (rDNAs) are present in multiple copies and are arranged in two families, the major rDNA (45S rDNA) and the minor rDNA (5S rDNA). The “nucleolus” 45S rDNA codes for 5.8S, 18S and 28S rRNA, which form the nucleolar organizer regions, NORs, and the “non-nucleolus” 5S rDNA codes for 5S rRNA [1].

The 45S rDNA sites are the most intensively studied, and as a result the most fully documented regions of chromosomes in various groups of eukaryotes. The eukaryotic genomes usually contain from hundreds to several thousand units of 45S rDNA. The latter are arranged in a series of “head-to-tail” tandem repeats, grouped on one, and sometimes on two or even more pairs of chromosomes and can be easily detected on chromosomes using fluorescence in situ hybridization (FISH). Data on the number and location of rDNA loci on chromosomes are actively used to study karyotype diversity, the organization of individual chromosomes, and the evolution of entire genomes. The above characteristics are also used as species-specific molecular genetic markers for studying evolution, taxonomy, and phylogeny, allowing for comparisons between taxa at different levels (see [2,3,4,5], and the references therein).

The insect suborder Heteroptera, or true bugs (order Hemiptera), consists of more than 42 thousand described species distributed in about 80 families and seven infraorders, including Enicocephalomorpha, Dipsocoromorpha, Gerromorpha, Nepomorpha, Leptopodomorpha, Cimicomorpha, and Pentatomomorpha [6,7,8].

To date, there is data on the chromosomal distribution of 45S ribosomal DNA for about 200 species of true bugs, which however belong only to the infraorders Nepomorpha, Cimicomorpha, and Pentatomomorpha ([9,10,11,12,13,14,15,16,17,18,19,20,21,22,23,24,25,26,27]; also see the references therein). In contrast, there is very little data on 5S rDNA in these insects [18,26,27].

As for 18S rDNA loci, certain patterns can already be traced in their chromosomal distribution in true bugs. FISH analyses of the species from different families have shown that 18S rDNA clusters are limited to from one to four per haploid genome, while their location is highly variable, especially in separate taxa (e.g., in the subfamily Triatominae of the family Reduviidae [14,15,16]). These clusters can be located on different types of chromosomes, including autosomes, the so-called m-chromosomes (a pair of specific very small chromosomes that occur in many heteropteran species and behave differently from both autosomes and sex chromosomes [28,29]); sex chromosomes; and in some species, both autosomes and sex chromosomes. Moreover, differences are sometimes observed even between closely related, congeneric species (reviewed in [13]). However, the most common feature of true bugs is the location of 18S rDNA on one pair of autosomes (found in half of the studied species [13]). This location has been recorded in species from different families, with different numbers of chromosomes and different sex chromosome systems (e.g., refs. [13,16,21,24]), and this feature is considered ancestral for the entire suborder Heteroptera [13]. In turn, although 18S rDNA clusters can be located at the terminal regions of the chromosome and interstitially within the chromosome, there is a clear tendency for them to be located near telomeres [13].

However, it is important to emphasize that species with known numbers and chromosomal positions of 45S rDNA clusters account for less than 0.5% of the described species of Heteroptera, with the vast majority of them belonging to Cimicomorpha and Pentatomomorpha. These sister infraorders, which include more than 16,000 species (in 40 families) and more than 20 thousand described species (in 16 families) worldwide, respectively, are the two major and most diverse infraorders constituting together the clade Terheteroptera, which is the terminal group of Heteroptera [7,30,31]. Thus, the available 18S rDNA data cover only a tiny fraction of the species diversity of Terheteroptera.

In this study, we obtained new data on the patterns of 18S rDNA arrangement in 13 species belonging to 13 genera and seven families of the infraorders Cimicomorpha and Pentatomomorpha. It is important to note that for each of the studied genera, such data were obtained for the first time. Our goal was to expand the understanding of the diversity of ribosomal gene localization in the chromosomes of these evolutionary lineages and the suborder Heteroptera as a whole.

## 2. Materials and Methods

The meiotic, though sometimes mitotic chromosomes of males of 13 species of true bugs belonging to seven families of the infraorders Cimicomorpha and Pentatomomorpha were analyzed. Table 1 shows the studied species, data, and collection sites, as well as the number of individuals studied.

The adult males were fixed immediately after collection in a fixative solution consisting of ethanol and glacial acetic acid (3:1) and stored in the fixative at 4 °C until slides were prepared. Chromosome preparations were obtained from the testes using the conventional squash method and were made permanent using the dry ice technique [32]. To study the standard karyotype of *H. graminis*, which was not previously known, the Schiff—Giemsa method [33] was used. To determine the distribution of 18S rDNA clusters in the chromosomes of each studied species, FISH was performed in accordance with a previously published protocol [13] using an 18S rDNA probe from gDNA of the red firebug *Pyrrhocoris apterus* (Linnaeus, 1758). In short, the target probe of 18S rDNA (a fragment about 1200 bp long) was amplified by PCR and labeled with biotin-11-dUTP (Fermentas, EU) using the specific primers 18S_F (5’-GATCCTGCCAGTAGTCATATG-3’) and 18S_R (5’-GAGTCAAATTAAGCCGCAGG-3’). The conditions for PCR analysis included an initial denaturation period of 3 min at 94 °C, followed by 35 cycles, each of which included denaturation for 30 s at 94 °C, annealing for 30 s at 55 °C, and extension for 110 s at 72 °C, with a final extension step of 3 min at 72 °C. The slides were treated with a solution containing 100 g/mL RNAse A and 5 mg/mL of pepsin to remove excess RNA and proteins from the DNA samples, washed sequentially in 1 × PBS, PBS × 1/0.05M MgCl_2_ and in 1% PFA in PBS × 1/0.05 M MgCl_2_, and then dehydrated in ethanol series. The chromosomes were denatured in a hybridization mixture containing labeled 18S rDNA probes with the addition of salmon sperm DNA to block non-specific binding in hybridization experiments, and then hybridized for 42 h at 37 °C. After standard washing procedures and blocking in 1.5% BSA/4 × SSC/0.1% Tween-20 [13], the 18S rDNA probe was detected using a NeutrAvidin-Fluorescein conjugate (Invitrogen, Karlsbad, CA, USA). The chromosomes were mounted in an antifade medium (ProLong Gold antifade reagent with DAPI, Invitrogen) and covered with a glass coverslip. At least 30 chromosome plates were examined on each slide. The most informative plates were selected for imaging.

The fluorescence images were acquired using a Leica DFC 345 FX camera (Leica Microsystems GmbH, Wetzlar, Germany) mounted on a Leica DM 6000B microscope (Leica Microsystems GmbH, Wetzlar, Germany) equipped with a 100x objective lens. The images were processed using the Leica Application Suite software version 4.5.0, which includes an Image Overlay module.

## 3. Results and Discussion

The studied material covers 13 species belonging to the infraorders Cimicomorpha (2 species) and Pentatomomorpha (11 species). Table 2 shows the diploid numbers of chromosomes (2n) with the karyotype formulas (number of autosomes and mechanism of sex chromosomes) and the results of physical mapping of 18S rDNA for each species included in this study, as well as previously published data on the chromosome numbers of the studied species. In all but one case, the diploid number of chromosomes and the karyotype formula were determined based on the stages of meiosis, most often prophase or metaphase I (MI), and mitotic chromosomes were studied only in *C. spiniger* (Coreidae).

**Figure 1:**
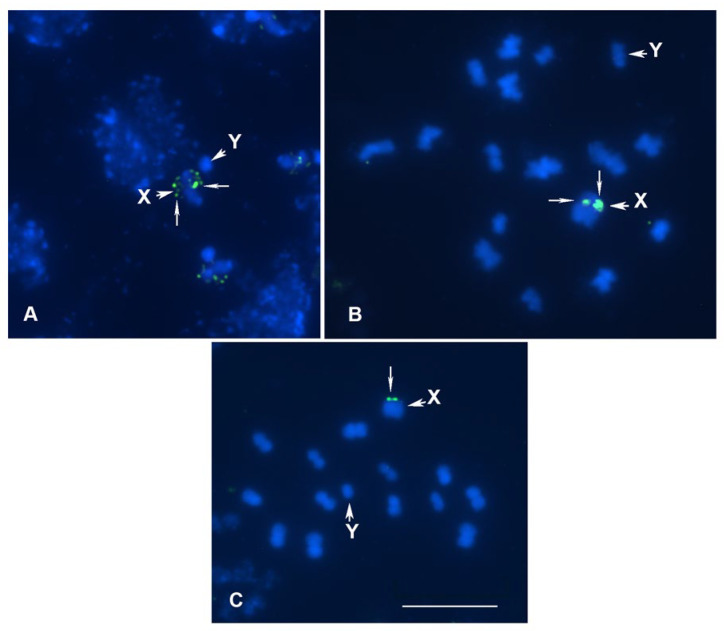
(**A**–**C**) Meiotic karyotypes of 3 species of Cimicomorpha (Miridae) after FISH with 18S rDNA probe. (**A**,**B**) *Horistus orientalis* early (**A**) and late (**B**) prophase condensation stages; (**C**) *Mimocoris rugicollis* MI. rDNA loci are indicated by thin arrows; X and Y—sex chromosomes. Bar = 10 µm in all figures.

**Chromosomal mapping of 18S rDNA clusters**
**Infraorder Cimicomorpha**
**Superfamily Miroidea**
**Family Miridae**
**Subfamily Mirinae**
***Horistus orientalis***, **meioformula: n = 16AA + X + Y** (Figure 1A,B)

**Karyotype.** In the studied males, 16 bivalents along with a pseudobivalent of the sex chromosomes, X and Y, were found at the condensation stage of meiotic prophase (Figure 1B), indicating a diploid karyotype with 34 chromosomes and the formula 2n = 32A + XY. Chromosomes are holokinetic, as in all Heteroptera [29], which means that they lack a localized centromere. Meiosis is clearly achiasmate, that is it lacks chiasmata, and consequently genetic crossover. However, homologous chromosomes in bivalents are connected to each other in one or more sites by tenacious threads known as collochores (the term was proposed in [41]), which ensures their pairing and segregation at anaphase [42]. A similar karyotype and “collochore meiosis” in this species were previously described in [34].

**18S rDNA location**. In the work of Grozeva and co-authors [34], FISH was performed on male meiotic chromosomes using an 18S rDNA probe, which detected a signal on the sex pseudobivalent, but its exact localization (on X or on Y) was not determined. According to our observations, the rDNA site is located on the X chromosome, which is clearly seen in Figure 1A, showing an early stage of spermatocyte meiosis with scattered signals on the X chromosome. This is the first clarifying data for the small plant bug genus *Horistus* Fieber, 1860, which includes only five species [43].

***Mimocoris rugicollis***, **meioformula: n = 13AA + X + Y** (Figure 1C)

**Karyotype.** The karyotype of this species has been studied for the first time. Moreover, this is the first data for the small genus *Mimocoris* J. Scott, 1872, which contains only two South Palearctic species [44,45]. In the studied males, 13 bivalents were found along with the X chromosome (large) and Y chromosome (small) as univalents at MI of meiosis, indicating 2n = 28 (26A + XY). The bivalents are similar in size, which makes it difficult to distinguish them from each other, which is typical for the family Miridae as a whole [34]. It is known that mirid bugs are characterized by the presence of an XY sex chromosome system and a high number of autosomes, more often 30-32 in a diploid set, and a karyotype with 26 autosomes, as in *M. rugicollis*, is very rare, including in the subfamily Mirinae [46], to which this species belongs. 

**18S rDNA location**. As in *H. orientalis*, the 18S rDNA cluster in *M. rugicollis* was located on the X chromosome; its position in this species was terminal.

**Figure 2:**
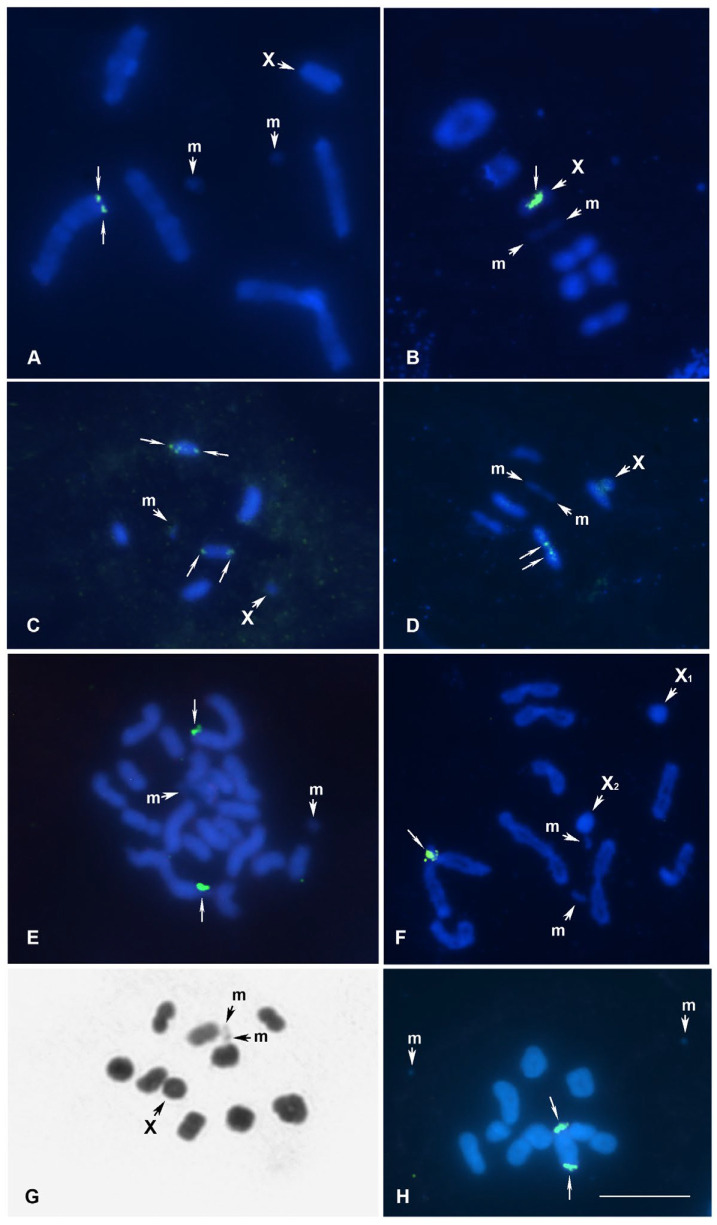
(**A**–**H**) Meiotic (except **E**) karyotypes of 6 species of Pentatomomorpha from families Rhopalidae (**A**–**D**) and Coreidae (**E**–**H**) after FISH with 18S rDNA probe (**E**,**F**,**H**) and after Shiff-Giemsa staining (**G**). (**A**) *Brachycarenus tigrinus* MI; (**B**) *Corizus hyoscyami* MI; (**C**) *Liorhyssus hyalinus* MI (only one m-chromosome is visible); (**D**) *Stictopleurus abutilon* MI; (**E**) *Centrocoris spiniger* spermatogonial mitotic plate, X chromosome is undistinguishable from autosomes; (**F**) *Coreus marginatus*, diplotene; (**G**,**H**) *Homoeocerus graminis* early MI. rDNA loci are indicated by thin arrows; X—sex chromosome, m—microchromosomes. Bar = 10 µm in all figures.

**Infraorder Pentatomomorpha**
**Superfamily Coreoidea**
**Family Rhopalidae**
**Subfamily Rhopalinae**
***Brachycarenus tigrinus***, **meioformula: n = 5AA + 2m + X** (Figure 2A)

**Karyotype.** Five autosomal bivalents, a pair of very small, negatively heteropycnotic m-chromosomes (as univalents), and an X chromosome were found at MI of the studied males, indicating 2n = 13(10A + 2m + X). The bivalents differ slightly from each other in size, and a similar karyotype was recently described for this species [35]. Moreover, this karyotype structure appears to be characteristic of the Rhopalidae family as a whole [29,47].

**18S rDNA location.** The 18S rDNA cluster is located on one of the largest bivalents, most likely AA1, and its localization is clearly terminal. This is the first data for the plant bug genus *Brachycarenus* Fieber 1860, which contains only two species [48].

***Corizus hyoscyami***, **meioformula: n = 5AA + 2m + X** (Figure 2B)

**Karyotype.** As in the previous species, *B. tigrinus* (Figure 2A), five autosomal bivalents, a pair of small, negatively heteropycnotic m-chromosomes (as a pseudo-bivalent), and an X chromosome were found at MI of the studied males, indicating 2n = 13(10A + 2m + X). Previously, the same karyotype was published for this species by other authors (see [29], and the references therein), including recently by Kuznetsova and co-authors [35].

**18S rDNA location** Using FISH mapping, we identified a large cluster of 18S rDNA on the X chromosome; its location on the chromosome remains uncertain. This is the first data to be found for the plant bug genus *Corizus* Fallén, 1814, which contains seven species [48].

***Liorhyssus hyalinus***, **meioformula: n = 5AA + 2m + X** (Figure 2C)

**Karyotype.** As in two previous species, seven elements were found in the haploid karyotype of this species, including five autosomal bivalents slightly differing in size, a pair of very small, negatively heteropycnotic m-chromosomes (a single m-chromosome is visible in Figure 2C), and an X chromosome, indicating 2n = 13(10A + 2m + X). This coincides with the data of the authors who previously studied the karyotype of *L. hyalinus* (see [29], and the references therein).

**18S rDNA location**. Using FISH mapping, we identified 18S rDNA loci in the terminal position on two bivalents, most likely AA2 and AA3. This is the first data to be found for the plant bug genus *Liorhyssus* Stål, 1870, which contains 12 species [48].

***Stictopleurus abutilon***, **meioformula: n = 5AA + 2m + X** (Figure 2D)

**Karyotype.** As in three previous species, seven elements were found in the haploid karyotype of this species, including five autosomal bivalents slightly differing in size, a pair of small, negatively heteropycnotic m-chromosomes, and an X chromosome, indicating 2n = 13(10A + 2m + X). Previously, Xavier [49] described the haploid karyotype of *S. abutilon* by the formula n = 5AA + mm + X, which coincides with our observations.

**18S rDNA location**. We found that the largest bivalent, AA1, carried terminally located rDNA sites in every homologue. This is the first data to be found for the plant bug genus *Stictopleurus* Stål, 1872, which contains 24 species worldwide [48].

**Family Coreidae**
**Subfamily Coreinae**
***Centrocoris spiniger***, **meioformula: 2n = 18A + 2m + X** (Figure 2E)

**Karyotype.** In this species, we have studied only mitotic chromosomes. Twenty-one chromosomes were found in spermatogonial mitoses, including 18 autosomes that vary slightly in size (however, the largest pair could be easily identified), a pair of very small m-chromosomes showing negative heteropycnosis, and the X chromosome (which is of medium size and difficult to identify among autosomes of a similar size), indicating 2n = 21(18A + 2m + X). Previously, the same chromosome number and the same karyotype formula were described for this species based on the study of meiosis [49,50].

**18S rDNA location**. Using FISH mapping, we identified 18S rDNA loci in the largest pair of autosomes (AA1), and their location was terminal in each homologue. This is the first data to be found for the genus *Centrocoris* Kolenati, 1845, which contains only nine valid species [48,51].

***Coreus marginatus***, **meioformula: n = 9AA + 2m + X1 + X2** (Figure 2F)

**Karyotype**. At the stage of diplotene of spermatocyte meiosis, the studied males had nine autosomal bivalents, as well as two univalent m-chromosomes and two sex chromosomes, X_1_ and X_2_, indicating 2n = 22 (18A + 2m + X_1_X_2_). The same karyotype was described for this species by Nokkala [36]. It should also be mentioned that many authors have studied the karyotype of *C. marginatus* repeatedly; in some cases, the authors describe the same karyotype, while in others they do not (see [29]).

**18S rDNA location**. Using FISH mapping, we identified 18S rDNA loci on the largest autosomal pair (AA1), and their location was terminal in each homologue. This is the first data to be found for the genus *Coreus* Fabricius, 1794, which contains only two species [48].

***Homoeocerus graminis***, **meioformula: n = 9AA + 2m + X** (Figure 2G,H)

**Karyotype.** We studied the karyotype of this species for the first time. During early spermatocyte MI, we observed nine bivalents of autosomes, as well as two univalent m-chromosomes and an X chromosome, indicating 2n = 21(18A + 2m + X). At this stage, the m-chromosomes either formed a bivalent (Figure 2G) or appeared as univalents (Figure 2H). The autosomal bivalents formed a decreasing size range, with three apparently larger ones.

**18S rDNA location**. The rDNA loci are located on one of the largest bivalents, presumably A1, and their location was terminal in each homologue (Figure 2H). This is the first data to be found on the genus *Homoeocerus* Burmeister, which contains 126 species [48].

**Figure 3:**
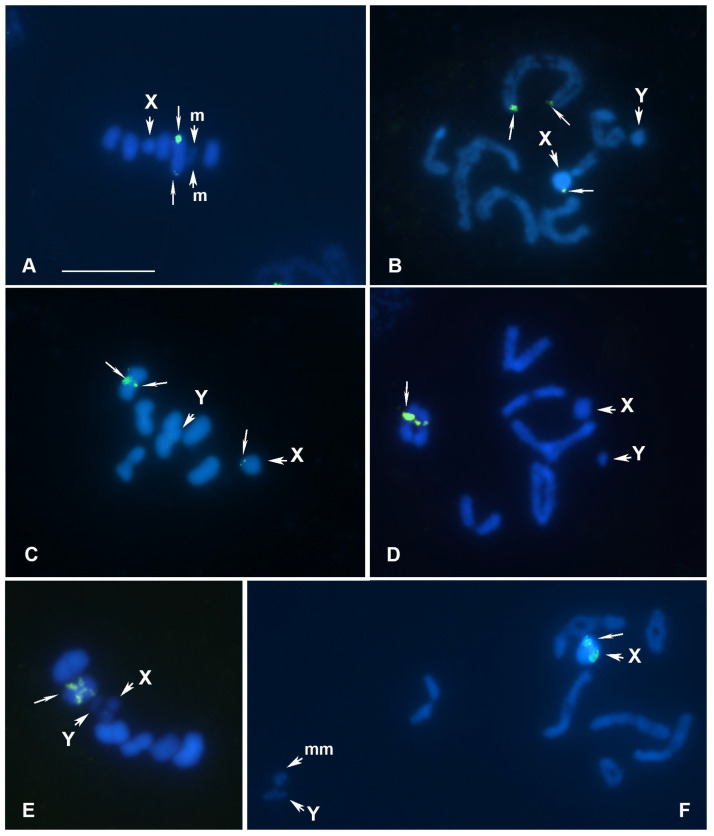
(**A**–**F**) Meiotic karyotypes of 4 species of Pentatomomorpha from families Stenocephalidae (**A**), Pentatomidae (**B**,**C**), Lygaeidae (**D**,**E**), and Rhyparochromidae (**F**) after FISH with 18S rDNA. (**A**) *Dicranocephalus agilis* MI; (**B**,**C**) *Piezodorus hybneri* diplotene (**B**) and MI (**C**); (**D**,**E**) *Lygaeus equestris* diplotene (**D**) and MI (**E**); (**F**) *Pterotmetus staphyliniformis* diplotene. rDNA loci are indicated by thin arrows; X and Y—sex chromosomes, m—microchromosome, mm—a pseudo-bivalent of m-chromosomes. Bar = 10 µm in all figures.

**Family Stenocephalidae**
***Dicranocephalus agilis***, **meioformula: n = 5AA + 2m + X** (Figure 3A)

**Karyotype.** At MI of the studied males, five bivalents of autosomes, two m-chromosomes forming a pesudobivalent at this stage, and X chromosome were observed, indicating 2n = 13(10A + 2m + X). The bivalents make up a decreasing size series, with one of them clearly larger than other ones (Figure 3A). Previously, Lewis and Scudder [37] studied the karyotype of *D. agilis*. They described the same karyotype and showed that one of the autosomal bivalents is associated with a nucleolus (NOR) at a “diffuse stage before diplotene”.

**18S rDNA location**. We detected rDNA-FISH signals on the largest pair of autosomes (AA1) in this species, which is consistent with the observations of Lewis and Scudder [37] mentioned above. The signals were located terminally in each homologue. The intensity of fluorescence signals differed between these loci, indicating that they had different copy numbers of the repetitive rDNA unit (Figure 3A). These rDNA data are the first for the family Stenocephalidae, which contains about 30 species in the single genus *Dicranocephalus* Hahn, 1826 [52].

**Superfamily Pentatomoidea**
**Family Pentatomidae**
**Subfamily Pentatominae**
***Piezodorus hybneri***, **meioformula: n = 6AA + X+Y** (Figure 3B,C)

**Karyotype.** At diplotene (Figure 3B) and MI (Figure 3C) of male meiosis, six autosomal bivalents plus X and Y sex chromosomes were observed, indicating 2n = 14(12A + XY). The bivalents form a series decreasing in size, in which we can easily identify the largest pair (AA1); of the two sex chromosomes, X is clearly larger than Y. Previously, the same karyotype was described for this species, including 14 chromosomes in a diploid set [38,39].

**18S rDNA location**. The rDNA loci are located terminally on the AA1 pair and on the X chromosome (Figure 3B,C). As in the previous species, the strength of fluorescence signals differed between these loci on AA1. The data obtained are the first for the genus *Piezodorus* Fieber, 1860, which contains about 12 species [53].

**Superfamily Lygaeoidea**
**Family Lygaeidae**
**Subfamily Lygaeinae**
***Lygaeus equestris***, **meioformula: n = 6AA + X+Y** (Figure 3D,E)

**Karyotype.** At diplotene (Figure 3D) and MI (Figure 3E) of male meiosis, six autosomal bivalents as well as X and Y sex chromosomes were observed, indicating 2n = 14(12A + XY). The bivalents form a decreasing size range, with two larger bivalents (AA1 and AA2) easily identified, whereas bivalents AA3 and AA4 appear to be similar in size; X is clearly larger than Y. The karyotype of *L. equestris* has been repeatedly studied by various authors (a review is given in [29]), including most recently by Kuznetsova and co-authors [35], and in all publications, the same karyotype formula, 2n = 14(12A + XY), is given for this species.

**18S rDNA location**. According to our observations, the rDNA loci are located on one of the medium-sized bivalents, most likely AA3, and their location looks like an interstitial one (Figure 3D,E). The data obtained are the first for the genus *Lygaeus* Fabricius, 1794, which includes 55 extant species [54].

**Family Rhyparochromidae**
**Subfamily Rhyparochrominae**
***Pterotmetus staphyliniformis***, **meioformula: n = 7AA + 2m + X+Y** (Figure 3F)

**Karyotype**. At diplotene of spermatocyte meiosis (Figure 3F), we observed seven bivalents of autosomes, as well as two univalent m-chromosomes and two sex chromosomes, X and Y, indicating 2n = 18(14A + 2m + XY). The meiotic karyotype of this species has been studied previously by Pfaler-Collander ([40]: as *P. staphylinoides*, Lygaeidae, Rhyparochrominae), with the same chromosome number and karyotype formula.

**18S rDNA location**. According to our observations, the rDNA loci are located on the X chromosome, and their position remains uncertain (Figure 3F). The data obtained are the first for the small genus *Pterotmetus* Amyot & A. Serville, 1843, which includes only three species [54].

## 4. Conclusions

We have physically mapped 18S rDNA on chromosomes of 13 species belonging to 13 genera (one species per genus) and seven families of the infraorders Cimicomorpha (family Miridae) and Pentatomomorpha (families Rhopalidae, Coreidae, Stenocephalidae, Pentatomidae, Lygaeidae, and Rhyparochromidae). All the species (except for *Horistus orientalis*, see above), all the genera, including *Horistus* (but see above), *Mimocoris*, *Pilophorus*, *Brachicarenus*, *Corizus*, *Liorhyssus*, *Stictopleurus*, *Centrocoris*, *Coreus*, *Homoeocerus*, *Dicranocephalus*, *Piezodorus***,** *Lygaeus*, and *Pterotmetus*; and the entire family Stenocephalidae have been studied in this regard for the first time. The species studied showed a high degree of variability in the chromosomal distribution of 18S rDNA.

As mentioned in the Introduction, the rDNA distribution patterns we found illustrate much of the variation shown by Heteroptera as a whole. In total, we identified three main types of rDNA location: (1) on the autosomes, (2) on the X chromosome, (3) on the autosomes and on the X chromosome simultaneously. The first two types include variants. In the first case (identified only in Pentatomomorpha), the rDNA loci can be located on the largest pair of autosomes, AA1 (six species of the families Rhopalidae, Coreidae and Stenocephalidae), on one of the medium-sized pairs (one species of Lygaeidae), or on two at once pairs, AA1 + AA2/A3 (1 species of Rhopalidae). In the second case (identified in both Cimicomorpha and Pentatomomorpha), the rDNA loci are located on the X chromosome (two species of Miridae and one species of Rhyparochromidae). The latter type, AA1 + X, has been found only in *Piezodorus hybneri* (Pentatomidae, Pentatomomorpha). The 18S loci were located either closer to the telomeres or in the interstitial regions of chromosomes, but near-terminal locations were more common. The predominant distribution of rDNA in the terminal regions of chromosomes is also observed in other groups of true bugs [13,14,15,55]. With all the diverse patterns of chromosomal localization of 18S rRNA genes identified in our study, autosomal localization clearly prevails. Interestingly, the largest pair of autosomes is more often the carrier of rDNA, which was also noted in other groups of true bugs, for example, in the subfamily Triatominae from the Reduviidae family [15]. An analysis of the available literature shows that the tendency to preserve rDNA in autosomes is observed in many species and in some higher taxa of true bugs (e.g., refs. [13,16,17,20,23,24,55,56]).

In our study, the rDNA loci were found only on the X chromosome in four species, including both the studied species of the family Miridae, the subfamily Mirinae, *Horistus orientalis* and *Mimocoris rugicollis*, which at the same time have different karyotypes—2n = 34 and 2n = 26, respectively. The situation with the Miridae is interesting, since all three previously studied species of this family, *Deraeocoris ruber* (Linnaeus, 1758) and *D. rutilus* (Herrich-Schaeffer, 1838) from the subfamily Deraeocorinae and *Megaloceroea recticornis* (Geoffroy, 1785) from the monotypic genus *Megaloceroea* Fieber, 1858 (subfamily Mirinae), also have 18S rDNA loci on their sex chromosomes [12]. It can be assumed that there is a relationship between the ribosomal genes and the sex chromosomes in Miridae. Moreover, the family Nabidae (from the same infraorder Cimicomorpha), which has data on 13 species, appears to exhibit the same tendency toward the presence of 18S rDNA on sex chromosomes [19,33]. Thus, further studies of both families are needed to verify the above assumption.

Thus, our rDNA-FISH analysis is limited to only 13 species, which however belong to 13 genera for which such data were obtained for the first time. Overall, the results obtained expand our understanding of the patterns of 18S rDNA loci distribution in the chromosomes of the large infraorders Cimicomorpha and Pentatomomorpha and confirm the importance of ribosomal DNA in the reorganization of the genomes of true bugs as a whole.

## Figures and Tables

**Table 1:** Material used for chromosomal analysis.

Species	Number of Specimens Studied, Data and Collection Sites
**Miridae**	
*Horistus orientalis* (Gmelin, 1790)	1 male, 23 May 2018, Kresna Gorge, Bulgaria (41°45′45″ N, 23°10′9″ E)
*Mimocoris rugicollis* (A. Costa, 1853)	1 male, 23 May 2018, Kresna Gorge, Bulgaria (41°45′45″ N, 23°10′9″ E)
**Rhopalidae**	
*Brachicarenus tigrinus* (Schilling, 1829)	4 males, 2 August 2024, Teberda vic., Russia (43°27′22″ N, 41°43′36″ E)
*Corizus hyoscyami* (Linnaeus, 1758)	2 males, 8 August 2024, Teberda vic., Russia (43°27′22″ N, 41°43′36″ E)
*Liorhyssus hyalinus* (Fabricius, 1794)	2 males, 23 October 2020, Perga vic., Turkey (36°57′28″ N, 30°51′7″ E)
*Stictopleurus abutilon* Rossi, 1790	1 male, 20 August 2024, 20 km N Voronezh, Russia (51°48′60″ N, 39°21′43″ E)
**Coreidae**	
*Centrocoris spiniger* (Fabricius, 1781)	1 male, 23 May 2018, Kresna Gorge, Bulgaria (41°45′45″ N, 23°10′9″ E)
*Coreus marginatus* (Linnaeus, 1758)	3 males, 20 August 2024, 20 km N Voronezh, Russia (51°48′60″ N, 39°21′43″ E)
*Homoeocerus* (*Anacanthocoris*) *graminis* (Fabricius, 1803)	1 male, 2 November 2019, mount Popa, Myanmar (20°55′16″ N, 95°15′12″ E)
**Stenocephalidae**	
*Dicranocephalus agilis* (Scopoli, 1763)	1 male, 25 June 2021, Sevan vic., Armenia (40°33′42″ N, 44°58′4″ E)
**Pentatomidae**	
*Piezodorus hybneri* (Gmelin, 1790)	1 male, 31 October 2019, Pagan, Myanmar (21°10′16″ N, 94°51′36″ E)
**Lygaeidae**	
*Lygaeus equestris* (Linnaeus, 1758)	2 males, 20 August 2024, 20 km N Voronezh, Russia (51°48′60″ N, 39°21′43″ E)
**Rhyparochromidae**	
*Pterotmetus staphyliniformis* (Schilling, 1829)	1 male, 20 August 2024, 20 km N Voronezh, Russia (51°48′60″ N, 39°21′43″ E)

**Table 2:** Karyotypes and chromosomal location of 18S rDNA in 13 species of Terheteroptera.

Taxa	2n and Karyotype Formula *	Location of 18S rDNA	Published Data on Chromosome Numbers	
**CIMICOMORPHA**				
**Miroideа**				
**Miridae**				
**Mirinae**				
*Horistus orientalis*	34 (32A + XY)	X	[34] **	
*Mimocoris rugicollis*	28 (26A + XY)	X	Absent	
**PENTATOMOMORPHA**				
**Coreoidea**				
**Rhopalidae**				
**Rhopalinae**				
*Brachycarenus tigrinus*	13 (10A + 2m + X)	AA1	[35]	
*Corizus hyoscyami*	13 (10 + 2m + X)	X	[29], and references therein [35]	
*Liorhyssus hyalinus*	13 (10A + 2m + X)	AA2 and AA3	[29], and references therein	
*Stictopleurus abutilon*	13 (10A + 2m + X)	AА1	[29], and references therein	
**Coreidae**				
**Coreinae**				
*Centrocoris spiniger*	21 (18A + 2m + X)	A1 pair ***	[29], and references therein	
*Coreus marginatus*	22 (18A + 2m + X_1_X_2_)	AA1	[29], and references therein; [36]	
*Homoeocerus graminis*	21 (18A + 2m + X)	AA1	Absent	
**Stenocephalidae**				
*Dicranocephalus agilis*	13 (10A + 2m + X)	AA1	[37]	
**Pentatomoidea**				
**Pentatomidae**				
**Pentatominae**				
*Piezodorus hybneri*	14 (12A + XY)	AA1 and X	[38,39]	
**Lygaeoidea**				
**Lygaeidae**				
**Lygaeinae**				
*Lygaeus equestris*	14 (12A + XY)	AA (a medium-sized pair)	[29], and references therein	
**Rhyparochromidae**				
**Rhyparochrominae**				
*Pterotmetus staphyliniformis*	18 (14A + 2m + XY)	X	[40]	

Note: A—autosome, AA—autosomal bivalent, AA1—the largest bivalent, X and Y—sex chromosomes; * the formulas of diploid (2n) karyotypes were deduced from the haploid (n) chromosome complements (at prophase or MI) of the first division of meiosis, with the exception of *** *C. spiniger*, in which the mitotic chromosomes were studied; **—rDNA-FISH detected signals on the XY pseudo-bivalent.

## Data Availability

The original contributions presented in this study are included in this article. Further inquiries can be directed to the corresponding author.
